# Immediate faculty feedback using debriefing timing data and conversational diagrams

**DOI:** 10.1186/s41077-022-00203-6

**Published:** 2022-03-07

**Authors:** Andrew Coggins, Sun Song Hong, Kaushik Baliga, Louis P. Halamek

**Affiliations:** 1 Department of Emergency Medicine, Westmead Hospital, Sydney, NSW 2145 Australia; 2 Sydney Medical School, Westmead Hospital Block K, Level 6, Westmead Hospital, Sydney, NSW Australia; 3 Division of Neonatal and Developmental Medicine, Department of Pediatrics, Stanford University, Palo Alto, CA USA

**Keywords:** Interprofessional collaborative practice, Debriefing, Faculty development (simulation educator or technician)

## Abstract

**Background:**

Debriefing is an essential skill for simulation educators and feedback for debriefers is recognised as important in progression to mastery. Existing assessment tools, such as the Debriefing Assessment for Simulation in Healthcare (DASH), may assist in rating performance but their utility is limited by subjectivity and complexity. Use of quantitative data measurements for feedback has been shown to improve performance of clinicians but has not been studied as a focus for debriefer feedback.

**Methods:**

A multi-centre sample of interdisciplinary debriefings was observed. Total debriefing time, length of individual contributions and demographics were recorded. DASH scores from simulation participants, debriefers and supervising faculty were collected after each event. Conversational diagrams were drawn in real-time by supervising faculty using an approach described by Dieckmann. For each debriefing, the data points listed above were compiled on a single page and then used as a focus for feedback to the debriefer.

**Results:**

Twelve debriefings were included (µ = 6.5 simulation participants per event). Debriefers receiving feedback from supervising faculty were physicians or nurses with a range of experience (*n* = 7). In 9/12 cases the ratio of debriefer to simulation participant contribution length was ≧ 1:1. The diagrams for these debriefings typically resembled a fan-shape. Debriefings (*n* = 3) with a ratio < 1:1 received higher DASH ratings compared with the ≧ 1:1 group (*p* = 0.038). These debriefings generated star-shaped diagrams. Debriefer self-rated DASH scores (µ = 5.08/7.0) were lower than simulation participant scores (µ = 6.50/7.0). The differences reached statistical significance for all 6 DASH elements. Debriefers evaluated the ‘usefulness’ of feedback and rated it ‘highly’ (µ= 4.6/5).

**Conclusion:**

Basic quantitative data measures collected during debriefings may represent a useful focus for immediate debriefer feedback in a healthcare simulation setting.

## Background

Providing adult learners with meaningful feedback is likely to be an important contributor to improved future performance [1–3]. Debriefing following simulation-based medical education (SBME) events is a key step in allowing participants to identify performance gaps and sustain good practice [3–5]. To achieve this goal, it is acknowledged that effective debriefing is important [6, 7]. Yet, as is often observed a gap may exist between ideal approaches to debriefing and actual performance [4].

To bridge this gap, a number of debriefing assessment tools provides a guide for rating and reviewing performance [8–10]. The tools available include the Objective Structured Assessment of Debriefing (OSAD) and the Debriefing Assessment for Simulation in Healthcare (DASH). OSAD and DASH assess debriefers on a Likert scale based on a set of ideal behaviours [8, 10]. As a result, they are useful for illustrating concepts to novices and providing a shared mental model of what a good debriefing looks like. However, they are not easily integrated into debriefer feedback, mentoring or coaching [11]. While these tools appear to be widely adopted in the training of debriefers, validation studies were limited to analysis of delayed reviews of recorded debriefings [8–10, 12]. In addition, while the tools may identify areas for the debriefer to improve, the arbitrary scores provided do not necessarily translate to improved future performance. In this study we seek to close this gap by exploring the use of quantitative data measures as a supplementary tool for debriefer feedback.

Current faculty development programmes often use the tools listed above as an aid to achieve improved debriefings [11]. In many programmes, feedback to new debriefers follows direct observation (or video review) by more experienced colleagues. Mentoring may also be useful if provided in a structured manner to help progress new debriefers towards mastery [13]. Coaching using a supportive and pre-agreed approach may also be important for facilitating stepwise improvements in debriefer performance [6, 13, 14]. These 3 strategies (i.e. feedback, mentoring and coaching) are attractive concepts but the best approaches to debriefer faculty development remain uncertain.

Based on an observation of debriefings conducted in various non-healthcare settings, we hypothesised that the use of quantitative data for feedback may provide an additional option for debriefer faculty development [15]. Notably, the current debriefing literature does not extensively report on using such quantitative data for debriefer feedback. There is a precedent for using a data-driven approach to feedback in healthcare more broadly [2, 15, 16]. Studies of data-driven feedback for healthcare providers showed improved team performance and this approach has been evaluated in both the social science and sporting literature [15–19].

As a result, in this study, we set out to (A) *examine the utility of basic quantitative debriefing performance data collected in real-time*; (B) *to compare the use of this data to existing assessment tools (i.e. DASH);* and (C) *to assess the future role of this approach for debriefer faculty development* [7, 20].

## Methods

### Study setting

The study was a collaboration between experienced debriefers at the Center for Advanced Pediatric and Perinatal Education (CAPE) at Stanford University (USA) and two Australian SBME centres in the Western Sydney Local Health District network [14]. This study explored the use of recording length of contributions during debriefings and use of conversational diagrams as a means of assessment of debriefing performance with reporting based on STROBE statement guidelines [21].

### Inclusion criteria and study subjects

Following the written consent of all simulation participants, debriefers and supervisors, we observed a series of 12 debriefings across two simulation sites. Debriefings were enrolled from January to March 2019 as a convenience sample selected on occasions where the availability of experienced supervising faculty as per the definition by Cheng et al. [13] allowed completion of the study protocols. At the time of data collection, COVID-19 pandemic social distancing restrictions were not in place. Observations and recording were conducted in real time for various elements using a paper data collection sheet. All the debriefings had a single lead debriefer and two supervising faculty present.

### Outcome measures

**Fig. 1:**
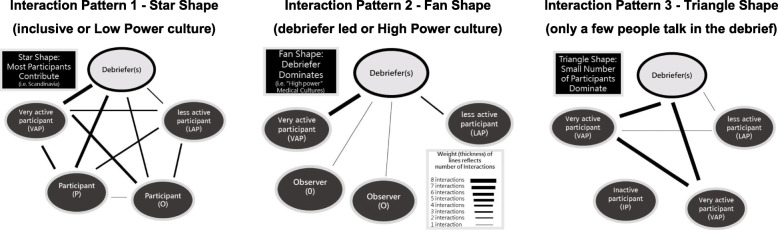
Conversational diagrams. Interaction and strength coding (adapted from Dieckmann et al. and Ulmer et al.). Interaction pattern 1—star shape (inclusive or low power culture). Interaction pattern 2—fan shape (debriefer led or high power culture). Interaction pattern 3—triangle shape (only a few people talk in the debrief)

We recorded the following data points in real time: (A) *study subject interactions* [7] (Fig. 1); (B) *timings*; (C) *quality* (DASH scores) [8] and (D) *demographics*. Demographics included role, gender and debriefing experience. Study subject age was not recorded. Junior doctors were defined as postgraduate year (PGY) 3 or less.

An a priori plan was made to assess the relationship between each member attending the debriefing by hand-drawing conversational diagrams for each debriefing (Fig. 1) [7]. The figures provided reflect the distribution of interactions, timing of each person and the relative strength of the interactions between each study subject. Two investigators observed each of the debriefings. Investigator A recorded the demographics of study subjects while Investigator B measured total time and the duration of conversation that each debriefing study subject contributed. Based on Dieckmann’s approach, we drew a line between two study subjects on the diagram who shared a strong interaction, which is defined as either a question and response or two connected statements in a debriefing [7]. In each resulting diagram, circles show each study subjects their roles and contribution timing, while the lines represent the significant interactions. As the study was in real time, we simplified diagram coding by not separating statements/questions exchanged between each person.

Utterances and gestures were not included in our scoring. Electronic diagrams presented were directly transcribed from free-hand drawn original diagrams. Any freehand or illegible annotations (*n* = 5) noted were excluded from the resultant electronic diagrams.

The timings of contributions of individual study subjects were measured using PineTree Watches™ Version 2.7.0 a multiple subjects stopwatch (www.pinetreesw.com). At the conclusion of each debriefing, Investigator A collected individual DASH scores from study subjects and completed the supervisor version of the scores [8].

### Debriefer feedback

Following each debriefing, semi-structured feedback was provided from supervising faculty. This was intentionally supplemented by referencing the data collection and was limited to 10 min. The approach used hybrisied the feedback methods described by Cheng et al. with the use of timing data and relational diagrams described above [14]. We assessed the impact by asking debriefers for a rating of the usefulness of the information provided (Likert scale 1–5).

### Analysis plan

Data were analysed using IBM SPSS (V24). Mean and standard deviation (SD) were used to summarise continuous variables. Frequencies and percentages were used for categorical variables. A two-sample t-test was used to test for differences in the distribution of continuous variables. A gestalt assessment of shape type (Fig. 1) was based on the work of Dieckmann et al. [7].

## **Res**ults

**Fig. 2:**
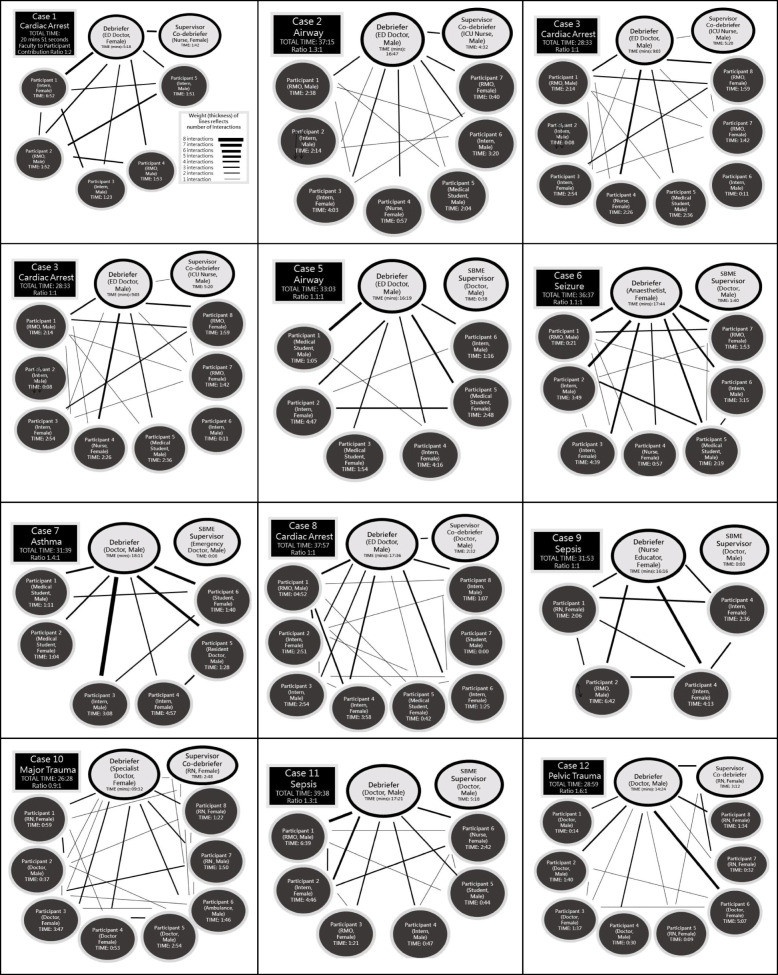
Timings and interactive diagrams of debriefings (*n* = 12)

Seventy eight simulation participants were enrolled comprising a mix of students (*n* = 14); doctors (*n* = 54); registered nurses (*n* = 9) and ambulance officers (*n* = 1). There was a high proportion (48.7%) of junior doctors and predominance of female subjects (53.8%). Baseline expertise of debriefers is outlined in Table 1 (divided into novice, intermediate, experienced) based on work by Cheng et al. [13]. The supervising faculty (*n* = 5) were all experienced based on Cheng's work. Figure 2 shows detailed contributions of all simulation participants, debriefers and supervising faculty combined with an illustrative representation of their interactions. The diagrams produced were a mixture of shapes (Fig. 1). In cases where debriefers talked for longer than the participants (ratio of ≧ 1:1), a fan-shaped appearance was typically observed. This shape is seen in cases 2, 5, 6, 7, 11 and 12 all of which had timing of contribution ratios suggesting relative debriefer ‘dominance’ (Fig. 2). Cases 1, 4 and 9 had a star-shaped appearance and all had a predominance of contributions from simulation participants (ratio of < 1:1). DASH Element 1 simulation participants’ ratings in the < 1:1 debriefings were higher than in the remaining (µ = 6.79 vs µ= 6.44; *p* = 0.036). None of the debriefings displayed a triangular shape, though we observed that students contributed less in large debriefings (i.e. cases 7, 8 and 11). Of note, nursing simulation participants appeared to contribute less to discussions than medical colleagues in the larger interdisciplinary debriefings (i.e. cases 10 and 12).

**Table 1:** Observational assessment of consecutive SBME debriefings

Topic	Study subject characteristics**	DASH scores					
		DASH element 1 Mean (SD)	DASH element 2 Mean (SD)	DASH element 3 Mean (SD)	DASH element 4 Mean (SD)	DASH element 5 Mean (SD)	DASH element 6 Mean (SD)
Case 1 Adult cardiac arrest	5 study subjects: 5 junior doctors (4 male; 1 female)	6.6 (0.49)	6.6 (0.49)	7 (0)	6.8 (0.4)	6 (0.63)	6.4 (0.49)
	1 debriefer: emergency registrar A, (male—novice*)	5	4	5	4	4	5
	1 supervisor: ICU nurse A (female—experienced*)	6	6	6	4	4	4
Case 2 Airway	7 study subjects: (5 junior doctors (3M; 2F), RN (F) student (F))	6.71 (0.45)	6.86 (0.35)	6.71 (0.45	6.57 (0.49)	6.43 (1.05)	6.57 (0.49)
	1 debriefer: emergency registrar B (female—novice*)	5	6	4	6	6	5
	1 supervisor: ICU nurse B (male—experienced*)	5	5	5	5	6	6
Case 3 Adult cardiac arrest	8 study subjects: (8 junior doctors (4M; 4F))	6.63 (0.70)	6.63 (0.70)	6.75 (0.43)	6.25 (0.97)	5.88 (1.45)	6.63 (0.48)
	1 debriefer: emergency registrar C (male—novice*)	4	5	6	5	5	6
	1 supervisor: ICU nurse B, (male—experienced*)	5	6	6	6	5	5
Case 4 Airway	5 study subjects: (5 junior doctors (3F; 2M))	6.8 (0.4)	6.8 (0.4)	7 (0)	7 (0)	7 (0)	7 (0)
	Debriefer: emergency registrar A (male—intermediate*)	5	5	5	4	5	5
	1 supervisor: specialist A (male—experienced*)	6	6	7	6	6	6
Case 5 Airway	6 study subjects: junior doctors (1M, 2F); 3 students (1M, 2F)	6.17 (0.37)	6.5 (0.5)	6.67 (0.47)	6.67 (0.47)	6 (1.15)	6.5 (0.5)
	Debriefer: emergency registrar A (male—intermediate*)	6	5	6	6	6	5
	1 supervisor: nurse educator A (female—intermediate*)	6	6	7	6	4	5
Case 6 Seizure	7 study subjects: 3 junior doctors (2M; 1F), 4 students (2M; 2F)	6.57 (0.49)	6.57 (0.49)	6.86 (0.35)	6.86 (0.35)	6.43 (0.73)	6.57 (0.49)
	1 debriefer: anaesthetic Doctor A (female—intermediate*)	5	5	4	5	5	5
	Supervisor: specialist A (male—experienced*)	6	6	7	6	4	5
Case 7 Asthma	6 Study Subjects: 3 Junior Doctors (2M; 1F), 3 Students (1M; 2F)	6.67 (0.47)	7 (0)	7 (0)	7 (0)	6.5 (0.5)	7 (0)
	Debriefer: Emergency Registrar A (Male - Intermediate*)	5	6	5	6	6	6
	Supervisor: Specialist (Male - Experienced*)	6	7	5	6	5	6
Case 8 Adult cardiac arrest	8 study subjects: 6 junior doctors (3M; 3F), 2 students (1M; 1F)	6.75 (0.43)	6.88 (0.33)	7 (0)	6.88 (0.33)	7 (0)	6.88 (0.33)
	Debriefer: emergency registrar C (male—intermediate*)	6	6	6	5	6	6
	Supervisor: specialist (male—experienced)	6	7	5	6	5	5
Case 9 Sepsis	4 study subjects: 3 junior doctors (1M; 2F), 1 RN (1F)	7 (0)	7 (0)	6.75 (0.43)	7 (0)	6.75 (0.43)	7 (0)
	Debriefer: nurse educator A (female—experienced*)	5	5	4	4	4	4
	Supervisor: specialist (male—experienced*)	6	6	d	6	6	6
Case 10 Trauma	8 study subjects: 4 doctor (2M; 2F), 3 RN (3F), 1 Ambu (M)	5.75 (0.43)	6.38 (0.48)	5.88 (0.60)	6.13 (0.78)	5 (1.22)	5.63 (0.86)
	Debriefer: emergency specialist A (female—experienced*)	4	5	5	4	5	5
	Supervisor: nurse (female—experienced*)	6	7	5	5	5	5
Case 11 Sepsis	6 study subjects: 4 doctor (2M; 2F), 1 RN (F), 1 student (M)	6.67 (0.47)	6.67 (0.47)	6.5 (0.5)	6.5 (0.76)	6 (1)	6 (1)
	Debriefer: emergency registrar B (male—intermediate*)	5	4	3	4	4	4
	Supervisor: specialist (male—experienced*)	6	6	5	6	6	5
Case 12 Trauma	8 study subjects: 5 doctor (3M; 2F), 3 RN (3F)	6.13 (0.60)	6.25 (0.66)	6.13 (1.05)	6.38 (0.86)	5.13 (1.45)	5.88 (0.93)
	Debriefer: emergency specialist B (male—experienced*)	5	6	5	6	5	5
	Supervisor: nurse (female—experienced*)	6	6	5	6	5	4
Overall DASH scores	Mean study subject DASH score (*n* = 78) (mean of total = 6.50)	6.50 (0.34)	6.64 (0.25)	6.66 (0.37)	6.63 (0.30)	6.12 (0.64)	6.46 (0.45)
	Mean self-debriefer DASH score (*n* = 12)(mean of total = 5.01)	5 (0.58)	5.17 (0.69)	4.83 (0.90)	4.92 (0.86)	5.08 (0.76)	5.08 (0.64)
	*p* value (*t* test)	*p* = < 0.0001	*p* = < 0.0001	*p* = < 0.0001	*p* = < 0.0001	*p* = < 0.0001	*p* = < 0.0001

*Footnote^13^: *< 10 Debriefings* = novice (‘discovery’), *10–50 Debriefings* = intermediate (‘growth’), *> 50 Debriefings* = experienced (‘maturity’)

DASH scores were provided by all simulation participants. For all six elements of the DASH scores, the debriefer self-assessments were much lower than the ratings provided by the participants. The differences reached statistical significance for all six DASH elements (*p* < 0.001). In regards to debriefers’ experience, of the 12 questionnaires shared 10 were returned resulting in a response rate of 83.3%. Debriefers rating the *‘usefulness’* of quantitative data provided for their feedback and indicated they found it useful (ų = 4.6/5 SD 0.49).

## Discussion

Debriefers have the challenging and rewarding task of guiding simulation participants in their post-experience reflection—both by affirming good behaviours and facilitating the remedy of shortfalls in performance [6, 22]. A debriefer’s ability to guide participants plays an important role in the delivery of simulation. In this observational study the striking findings included a predominance of debriefers talking more than participants (Fig. 2), significantly higher DASH scores provided by participants compared with those self-rated by debriefers and higher participant DASH scores for the debriefers who talked less. In addition, we observed a high level of debriefer satisfaction in using basic quantitative data (timing and diagrams) as an aid to providing feedback. We have structured the following discussion based on the three objectives outlined in the background section.

### Can real-time quantitative debriefing performance data be used for feedback?

This study assessed the use of timing data and conversational diagrams. Debriefers receiving feedback based on this data rated its *‘usefulness’* as ų = 4.6 on a 5-point Likert scale. This is an encouraging finding. While it does not guarantee translation into better debriefing, in other settings data-driven feedback has been shown to significantly improve performance [2, 23]. This study was interrupted by the recent COVID-19 pandemic leading to an under-recruitment of debriefings (*n* = 12), yet we were still able to observe a broad range of interdisciplinary simulation participants and 7 debriefers across 2 SBME sites (Table 1). This suggests that results can be extrapolated to other locations.

Regarding the use of timing data, we present the results for individual times and ratios of contributions of debriefers versus simulation participants (Fig. 2). While the timing data set is interesting within the boundaries of study conditions, it is unclear if this would be practical to measure in typical simulation environments due to resource constraints. It is also unclear whether individual timing data is useful to the debriefers receiving feedback or whether timings reflect quality. For example, knowing an individual talked for a certain period does not necessarily reflect the usefulness of the content, nor the appropriateness of the contribution for promoting reflection. Within these limitations, in using the data for feedback we found it easy to start meaningful conversations with the debriefers about improving future performance [14]. For example, the data on timing allowed discussion of how to involve quieter participants, and how to increase the number of questions that encouraged reflection rather than ‘guess what I am thinking’. While the availability of timings and diagrams appeared to help with feedback, this information arguably may also have been provided using direct observation alone.

From a practical standpoint, we recommend for measuring timing data that a chess clock would be sufficient. A chess clock can provide a simplified binary division of simulation participant and debriefer contributions and would be more practical than our tested method. This approach could provide an estimation of how much the debriefer is talking compared to the participants [6]. With this in mind, from the study findings we note that many debriefings appear to fit a *‘sage on the stage’* category. This is evidenced by 9/12 debriefings in which facilitators talked for equal or longer than the simulation participants. This important finding may be explained by the increasing requirement of multiple hats to be worn by simulation educators or by a lack of training in our debriefer cohort. Debriefers may revert into more familiar approaches to teaching during debriefings such tutoring, explanations and lecturing [24]. To address this problem, timing data could help shape future behaviour. Of interest, in a concurrent study we are also investigating the use of cumulative tallies of debriefer questions, debriefer statements and simulation participant responses. In a similar way to using the chess clock approach for timing, this approach may provide an easy to measure method of estimating the debriefer inclusivity.

In regard to the conversational diagrams, these illustrations were used concurrently with the timing data (Fig. 2) for feedback to debriefers. These diagrams were described by Dieckmann et al. in terms of typical roles in SBME, as well as Ulmer et al who described a variety diagram shapes according to culture [7, 20]. We divided the debriefings in terms of those where the debriefer(s) spoke more than or equal to simulation participants (*n* = 9) and events where the debriefer(s) spoke less (*n* = 3). Using this binary division as a basis for analysis, we observed a pattern in the corresponding shapes of the diagrams (Fig. 2). Similar appearances and shape patterns were reported in Dieckmann and Ulmer’s papers [7, 20]. However, on close inspection of each diagram obtained, we could not find the triangular pattern described by Dieckmann et al. The triangle pattern (Fig. 1) is suggestive of 2 or 3 participants dominating. An absence of this pattern was surprising as the range of contribution lengths varied widely (Fig. 2) with some participants not talking at all and some participants talking for > 6 min. This finding could be due to errors in diagram drawings or random chance. Future studies could avoid any uncertainty in this area by analysing debriefings carefully with use of video and professional transcription.

The astute reader would note that medical students contributed less in larger debriefings (i.e. cases 7, 8 and 11) and nurses contributed less than physicians in mixed groups (i.e. cases 10 and 12). This important observation reminds us of the importance of ensuring a simulation learning climate that feels safe for all, and that the topics chosen for discussion in the debriefing are of interest to all [25–27]. In this study, the majority of recorded interactions were between the debriefer and simulation participants. Very few interactions were recorded between the participants—an important omission—which may represent a target for our own approaches to faculty development at a local level.

In summary, the simulation literature outlines an array of behavioural characteristics exhibited by debriefers that can promote improved future performance [6]. Existing assessment tools such as DASH have an established role in identifying these characteristics. Use of timing data and conversational diagrams may represent an adjunct which may help debriefers understand their performance, track changes over time and assist supervisors in providing debriefer feedback.

### How does quantitative debriefing performance data compare to existing tools?

Existing debriefing assessment tools such as DASH have pros and cons that have been briefly described in the background section. In this study DASH scores were provided by all debriefers and simulation participants. While this was not the primary outcome, it shines a light on the limitations of DASH use. Of note, the 7 debriefers rated themselves significantly lower than the scores from the simulation participants for all DASH elements. These findings reflect our personal experience of using DASH. Prior to the study we had also observed debriefers underscoring themselves and simulation participants overscoring. This finding is interesting, and may be explained by the phenomenon of *‘response bias’*, which is reported as a problem of assessment scales and surveys [28, 29]. Variation in DASH scores between raters, as well as the time that DASH takes to complete, may reflect the relative subjectivity of the scores provided and limit its value for debriefer feedback [14]. Furthermore, neither the DASH nor OSAD provide specific measurable goals for new debriefers to target in their next debriefing. Therefore, we suggest a continued use of DASH for highlighting ideal behaviours with supplementation of the various quantitative data tools we have outlined in this paper.

### What is the potential role of these findings in the development of debriefers?

As stated, the recipe for success for debriefer faculty development may not come from a single approach. In this study, we found the availability of both quantitative and qualitative data was useful. Experience of using timing data and diagrams together was generally positive, but recording the data and applying this approach was resource intensive. Moreover, the recent pandemic has limited SBME in-person interactions, making current applicability questionable. In the context of the current remote learning climate, a recent study recognised that current methods of faculty development lack a structured approach [30]. We agree that structure is clearly an important factor that faculty development programmes might lack [11]. The quantitative approaches described in our work may assist with providing this structure at the point of care by allocating our attention to observing debriefings in a focused manner. The approaches described should not supercede local planning, adequately resourced and culturally sensitive debriefer faculty development [11, 30].

In terms of other solutions to a relative lack of structure in faculty development programmes, some experts have proposed the use of *DebriefLive*Ⓡ. This is a virtual teaching environment that allows any debriefer to review their performance [30]. Using this (or similar) software could allow debriefers to observe videos, rate themselves and track progress. In view of the current need for social distancing and the use of remote learning, video review may be an alternative to use of the paper forms and real-time feedback that we used [31–33].

## Limitations

The limitations of our findings are acknowledged especially in relation to the relatively small sample size of the study. We also accept that results aree context specific and the approaches described would prove challenging outside of a research setting. Regarding use of the DASH tool as a ‘gold standard’, we note that this tool has been through limited validation. The relevant study used 3 example videos that were scored remotely by online reviewers [8]. On the other hand, validation of OSAD was much broader with studies conducted on electronic versions and in languages other than English [12, 33, 34]. We acknowledge that it is possible our results would have been different had OSAD been used [10]. Regardless, it is our view that the use of any tool as a single approach to faculty development is limited. Locally, we are now using the tools listed above with the data-driven approach assessed in the study [35]. We use either video conferencing or a real-time approach depending on the current local policy on social distancing and remote learning [36].

In conclusion, the use of quantitative data alongside traditional approaches to feedback may be useful for both debriefers looking to improve their future performance and supervising faculty seeking to improve local faculty development programmes.

## Data Availability

No additional data available.

## Abbreviations

CAPE: Center for Advanced Pediatric and Perinatal Education
COVID-19: Coronavirus disease 2019
DASH: Debriefing Assessment for Simulation in Healthcare
ED: Emergency department
ICU: Intensive care unit
OSAD: Objective Structured Assessment of Debriefing
PGY: Postgraduate year
RN: Registered nurse
SBME: Simulation-based medical education
STROBE: Strengthening the Reporting of Observational studies in Epidemiology
WSLHD: Western Sydney Local Health District
