# Correction: Immediate faculty feedback using debriefing timing data and conversational diagrams

**DOI:** 10.1186/s41077-023-00247-2

**Published:** 2023-02-24

**Authors:** Andrew Coggins, Sun Song Hong, Kaushik Baliga, Louis P. Halamek

**Affiliations:** 1 Department of Emergency Medicine, Westmead Hospital, Sydney, NSW 2145 Australia; 2 Sydney Medical School, Westmead Hospital Block K, Level 6, Westmead Hospital, Sydney, NSW Australia; 3 Division of Neonatal and Developmental Medicine, Department of Pediatrics, Stanford University, Palo Alto, CA USA

**Correction: Advances in Simulation 7, 7 (2022)**

**https://doi.org/10.1186/s41077-022-00203-6**

The original article [1] contained an error in Fig. 2 whereby Case 3 was duplicated over Case 4; this error has since been rectified.

## References

[1] Coggins A, Hong SS, Baliga K, Halamek LP (2022). Immediate faculty feedback using debriefing timing data and conversational diagrams. Advances in Simulation.

